# Secondary Transcriptomic Analysis of Triple-Negative Breast Cancer Reveals Reliable Universal and Subtype-Specific Mechanistic Markers

**DOI:** 10.3390/cancers16193379

**Published:** 2024-10-02

**Authors:** Naomi Rapier-Sharman, Mauri Dobbs Spendlove, Jenna Birchall Poulsen, Amanda E. Appel, Rosana Wiscovitch-Russo, Sanjay Vashee, Norberto Gonzalez-Juarbe, Brett E. Pickett

**Affiliations:** 1 Department of Microbiology and Molecular Biology, Brigham Young University, Provo, UT 84602, USA; naomi.rapier.sharman@gmail.com (N.R.-S.); mauridobbs@gmail.com (M.D.S.); jennabirchall@gmail.com (J.B.P.); 2 Infectious Diseases and Genomic Medicine Group, J. Craig Venter Institute, Rockville, MD 20850, USA; aappel@jcvi.org (A.E.A.); rrusso@jcvi.org (R.W.-R.); ngj@umd.edu (N.G.-J.); 3 Synthetic Biology and Bioenergy Group, J. Craig Venter Institute, Rockville, MD 20850, USA; svashee@jcvi.org

**Keywords:** TNBC, transcriptional mechanistic marker, prognosis, RNA-sequencing, drug repurposing, CIDEC, ASPM, CD300LG, RGS1, TNMD

## Abstract

**Simple Summary:**

Breast cancer is diagnosed in 2.3 million women each year and kills 685,000 (~30% of patients) worldwide. The most dangerous breast cancer is triple-negative breast cancer (TNBC). TNBC is very diverse, with 12 underlying subtypes. This great deal of patient diversity, along with the lack of broad-application targetable mechanistic markers (such as ER, PR, and HER2, which are present in other breast cancer subtypes but missing in TNBC), gives TNBC patients the worst outcomes of any breast cancer type. We aim to remedy this by exploring the molecular mechanisms across TNBC samples and subtypes. Molecular mechanisms are potentially targetable for treatment. We explore these options as part of our RNA-sequencing analysis. Our novel findings include highly accurate mechanistic markers identified using machine learning methods, including CIDEC (97.1% accuracy alone). Additionally, we found TNBC subtype-differentiating mechanistic markers, including PDE3B, CFD, IFNG, and ADM, which are targets with known therapeutics and potential for drug repurposing.

**Abstract:**

**Background/Objectives**: Breast cancer is diagnosed in 2.3 million women each year and kills 685,000 (~30% of patients) worldwide. The prognosis for many breast cancer subtypes has improved due to treatments targeting the estrogen receptor (ER), progesterone receptor (PR), and human epidermal growth factor receptor 2 (HER2). In contrast, patients with triple-negative breast cancer (TNBC) tumors, which lack all three commonly targeted membrane markers, more frequently relapse and have lower survival rates due to a lack of tumor-selective TNBC treatments. We aim to investigate TNBC mechanistic markers that could be targeted for treatment. **Methods:** We performed a secondary TNBC analysis of 196 samples across 10 publicly available bulk RNA-sequencing studies to better understand the molecular mechanism(s) of disease and predict robust mechanistic markers that could be used to improve the mechanistic understanding of and diagnostic capabilities for TNBC. **Results:** Our analysis identified ~12,500 significant differentially expressed genes (FDR-adjusted *p*-value < 0.05), including KIF14 and ELMOD3, and two significantly modulated pathways. Additionally, our novel findings include highly accurate mechanistic markers identified using machine learning methods, including CIDEC (97.1% accuracy alone), CD300LG, ASPM, and RGS1 (98.9% combined accuracy), as well as TNBC subtype-differentiating mechanistic markers, including the targets PDE3B, CFD, IFNG, and ADM, which have associated therapeutics that can potentially be repurposed to improve treatment options. We then experimentally and computationally validated a subset of these findings. **Conclusions:** The results of our analyses can be used to better understand the mechanism(s) of disease and contribute to the development of improved diagnostics and/or treatments for TNBC.

## 1. Introduction

Breast cancer is the most common solid tumor type in women [1], resulting in over 2 million diagnoses and ~685,000 deaths annually [2,3]. Triple-negative breast cancer (TNBC) comprises 10–20% of breast cancer cases and is one of the most aggressive types of breast cancer [2,3]. TNBC is highly prevalent among premenopausal women under 40 and women of African or Hispanic descent [4,5]. The presence of a BRCA1 or BRCA2 mutation is a risk factor, and at least one of these mutated loci is present in as many as 19.5% of TNBC cases [5]. TNBC is often compared to basal-like breast cancer (BLBC) since both of these cancer types share approximately 56% of their gene expression profile [6]. TNBC, by definition, lacks the three typical surface markers and is thus refractory to common therapeutic targets of breast cancer, including the estrogen receptor (ER), progesterone receptor (PR), and human epidermal growth factor receptor 2 (HER2), which severely limits its therapeutic options and justifies continued efforts to develop additional TNBC-specific treatments.

Current pharmacological treatments for non-TNBC breast cancers include tamoxifen, a competitive inhibitor of the estrogen receptor; aromatase inhibitors, which target the enzyme that performs the last step of estrogen biosynthesis; and trastuzumab, which binds the HER2 receptor [7,8]. Unfortunately, the lack of the three hallmark extracellular receptors that are targets for other types of breast cancers, ER, PR, and HER2, severely reduces the number of available therapeutic options for TNBC patients [9]. TNBC is accompanied by an increased risk of metastasis after neoadjuvant chemotherapy, defined as chemotherapy followed by surgical extraction of the tumor, while the post-surgical recurrence rate of TNBC can be as high as 25% [10]. The poor prognosis and low success rate of surgical treatments and chemotherapeutic options emphasize the necessity of early diagnosis and better treatments, especially since the five-year mortality rate for TNBC patients is ~40% [6].

The early detection of TNBC, typically diagnosed by immunohistochemistry and/or imaging [11,12], leads to more positive patient outcomes and higher patient survival rates [13]. Furthermore, a careful analysis of the gene expression, predicted biomarkers, and mechanistic markers, as well as the signaling pathway profile of TNBC, can facilitate the identification of therapeutic targets and their associated therapeutics that can be repurposed for TNBC [14,15,16]. The aim of the current study was to perform a joint secondary analysis of 196 publicly available clinical bulk RNA-sequencing samples to improve the confidence in the differentially expressed gene (DEG) results [15,17]. We then used the RNAseq data to calculate mechanistic pathways and to predict robust mechanistic markers that could aid in the development of improved diagnostic capabilities.

## 2. Materials and Methods

### 2.1. Data Collection

Public RNA-sequencing (RNA-seq) data from collected clinical material were acquired from the National Center for Biotechnology Information (NCBI) Gene Expression Omnibus (GEO) database using the search terms “triple negative breast cancer” and “breast”, with the goal of finding breast tumor samples and tissue-specific healthy controls [18]. All 297 studies matching the phrase “triple negative breast cancer” that were available as of 19 October 2022 were manually reviewed, as were 1356 other studies that were retrieved using the “breast” query to increase the number of tissue-specific control samples. Specifically, the query results were filtered by including criteria for samples collected from humans and “Expression profiling by high-throughput sequencing”. To minimize the downstream impact of irrelevant data, RNA-seq samples from cell lines, formalin-fixed paraffin-embedded tissues, gene expression microarray experiments, single-cell RNA-sequencing experiments, xenografts, metastases, adjacent normal tissues, sequencing performed on platforms other than Illumina, and more diverse tissue types (whole blood, PBMCs, brain, etc.) were manually identified and excluded. While a subset of healthy control samples was obtained from the same studies as the cancer samples, other healthy control samples were obtained from other studies to create roughly equivalent-sized cancer and healthy groups. The final dataset that was assembled from GEO for a secondary analysis consisted of 196 samples (109 TNBC samples and 87 healthy breast tissue samples) from 10 studies [19,20,21,22,23,24,25,26,27,28,29,30,31]. The raw data for these experiments were previously collected under appropriate ethical oversight to protect patient autonomy and patient identity (Supplementary Figure S1A–C and Table 1).

### 2.2. Preprocessing of RNA-Sequencing Data

Following the manual curation of the RNA-seq samples, FASTQ files were preprocessed as previously described [32,33,34,35]. In brief, the FASTQ files containing RNA-sequencing data were downloaded from the Sequence Read Archive (SRA) using sra-tools (version 2.10.8). The FASTQ files, the associated metadata file (Supplementary File S1), and a configuration file were used as inputs to the snakemake-based Automated Reproducible MOdular workflow for preprocessing and differential analysis of RNA-seq data (ARMOR; version 1.5.7) [36,37]. More specifically, the ARMOR workflow includes the following steps: trimming sequencing adapters and poor-quality regions of the reads with TrimGalore! [38], calculating quality control metrics with FastQC [39], pseudomapping and quantifying reads to the human GRCh38 transcriptome (release 98) with Salmon (version 1.3.0) [17], calculating significant differential gene expression using edgeR (version 3.36.0) [40], and performing Gene Ontology (GO) enrichment against terms in the MSigDB [41] while adjusting for inter-gene correlation using the Camera algorithm (version 3.50.3) [42].

### 2.3. TNBC Subtype Prediction

Following read mapping using Salmon (version 1.3.0) [17], transcript-level read counts were compiled into a tabular format using a custom script, as previously reported [43], prior to predicting the TNBC subtype for each sample using the TNBCtype algorithm (accessed August 11, 2023) [44]. These predicted subtypes and read count data were used for subsequent analyses, including cluster visualization using a principal component analysis (PCA) [45] and the prediction of universal TNBC and TNBC subtype-specific transcriptional mechanistic markers.

### 2.4. Mechanistic Marker Prediction Using Read Count Data

As stated above, the transcript-level read counts, generated by Salmon (version 1.3.0), were organized into a tabular format, and the samples were randomly assigned to either a training set (80%) or a testing set (20%). The XGBoost (version 1.7.7.1) library in R was then used in a custom code to run a supervised tree-based classification analysis, with disease state (healthy or TNBC) as the classification label, to predict the most robust features/mechanistic markers using the gain metric [46,47]. The initial results from the whole transcriptome were then reduced to the best five upregulated and the best five downregulated transcriptional mechanistic markers based on the gain metric for each of the features. The highest-ranked mechanistic markers were then tested for their predictive power against the entire dataset and individual subtypes. The area under the curve (AUC) was calculated from the receiver operator characteristic (ROC) curves that were generated for each set of mechanistic marker predictions to determine the overall performance of the selected mechanistic markers for distinguishing between TNBC and healthy tissue. Additionally, subtype-specific mechanistic markers were predicted using a similar approach to that described above based on the subtypes generated by the TNBCtype algorithm (accessed August 11, 2023) for each of the samples included in the dataset. For the subtype-specific mechanistic marker classifications, the samples belonging to each subtype were predicted using the tree-based method in XGBoost, with the same percentage of samples randomly assigned to either the testing or training sets. The samples belonging to each TNBCtype classification were assigned the “TNBC” label to identify the features that are the most useful in classifying each subtype.

### 2.5. Additional Analysis of Differentially Expressed Genes

To adhere to the accepted transparent reporting of joint secondary analysis generation and results, a PRISMA flowchart was generated (Supplementary Figure S1A) [48]. The differential expression and mechanistic marker data were combined and represented as a heat map using the R package ggplot2 (version 3.5.0) and a custom script [49,50]. The gene read count values for the 20 best features predicted by XGBoost (version 1.7.7.1) and the 20 most significant DEGs across all samples were extracted from the Salmon files and normalized with a log base-2 adjustment and z-score normalization by either sample or gene to reduce the effects of extreme outliers on the visualization. The STRING protein–protein interaction database was used to investigate the annotated connections between the gene products from the top 20 DEGs and top 20 predicted transcriptional mechanistic markers [51]. Connections between KIF family members and the 2022 list of American College of Medical Genetics and Genomics (ACMG) cancer-associated genes were also generated using annotations in the STRING database [52].

### 2.6. Drug Prediction Using Modulated Pathways and Predicted Mechanistic Markers

The significant differentially expressed genes from the ARMOR workflow were then used as input to the Signaling Pathway Impact Analysis (SPIA; version 2.50.0) algorithm to enrich differentially expressed genes against intracellular signaling pathways from five databases, namely, KEGG, Panther, BioCarta, Reactome, and NCI [53,54,55,56,57]. The differentially expressed genes that were output by ARMOR were evaluated by the log2 fold change and false discovery rate-adjusted *p*-value (FDR *p*-value < 0.05). The threshold for significant pathways using the SPIA algorithm (version 2.50.0) was Bonferroni-adjusted *p*-values < 0.05. Pathways that were predicted to be significantly modulated by the SPIA algorithm (version 2.50.0) were used to predict targets and existing therapeutics that affect those targets, which could be candidates for repurposing against TNBC, using the Pathway2Targets algorithm (version 2.2) [14]. Briefly, the Pathway2Targets algorithm (version 2.2) takes the significantly affected pathways, retrieves the protein members of those pathways, searches the Open Targets drug database [58] for therapeutics known to target the proteins in each pathway, and ranks drug targets by their relevance to the disease according to ~10 attributes. Default target prioritization scoring parameters were used for Pathway2Targets to prioritize targets present in multiple pathways, targets implicated in a high number of associated diseases, and targets with a higher number of therapeutics further along in clinical trials. An altered form of the Pathway2Targets script was also used to retrieve drug availability data for all predicted mechanistic markers presented in the results tables [59].

### 2.7. In Vitro TNBC RNA-Seq Analysis

RNA-seq composite expression data for the NCI-60 cell line collection were downloaded from the CellMiner database (version 2.11) on 1 February 2024 [60,61,62]. The composite RNA-seq expression data from the three TNBC cell lines in the NCI-60 cell line collection (i.e., BT-549, HS 578T, and MDA-MB-231) were isolated for further analysis. In brief, the CellMiner TNBC cell line data, presented in units of Fragments Per Kilobase per Million reads on a log2 scale (log2(FPKM + 1)), were converted back to raw FPKM. Zeroes in the CellMiner table fields were replaced with “NA” to indicate non-detection in the CellMiner dataset before FPKM conversion into units of Transcripts Per Million (TPM) using Harold Pimentel’s conversion equation: TPM = exp(log(FPKM)—log(sum(FPKM)) + log(1 × 10^6^) [63]. The TPM values for the primary TNBC dataset discussed in this paper were extracted from the Salmon files. Log2 fold change values were generated for each detected gene, using the genes’ mean TPMs from the healthy primary samples as the denominator, to allow for an effective comparison between the cell line and primary TNBC data. Genes that had 0 TPM in the CellMiner database samples were noted in the output table as not detected (ND).

### 2.8. In Vitro Immunoblots

Whole-cell extracts were prepared with a RIPA buffer (150 mM NaCl, 1% NP-40, 0.5% DOC, 0.1% SDS, and 50 mM Tris) containing protease inhibitors (Sigma, St. Louis, MO, USA). The samples were loaded into 4–15% gradient gels at 20 mg per lane, separated by SDS-PAGE, and transferred to nitrocellulose membranes. The membranes were blocked in a blocking buffer (TBS-0.01% Tween-20 containing 5% BSA) for at least 1 h. Primary antibodies were diluted in the blocking buffer, and the membranes were incubated overnight at 4 °C. The primary antibodies used included anti-KIF14 antibody (A10275, ABclonal, Woburn, MA, USA), anti-TNMD antibody (A17753, ABclonal), anti-MTHFD1L antibody (A7969, ABclonal), and β-actin as a loading control (ab8226, Abcam, Waltha, MA, USA). HRP-tagged secondary antibodies were used to detect the primary antibodies (1:5000 in blocking buffer) for 1 h. SuperSignal West PICO Plus (Product 34580, ThermoFisher, Waltham, MA, USA) was used to develop HRP. All images were collected on an Amersham Imager 680 (GE) and analyzed for densitometry in ImageJ briefly to quantify the protein bands in Western blots relative to the β-actin loading control.

## 3. Results

### 3.1. Search of Gene Expression Omnibus Yields 196 Bulk RNA-Seq TNBC Samples

We began by acquiring the relevant samples from the NCBI Gene Expression Omnibus (GEO) database. Specifically, we searched for studies that generated RNA-seq data from human breast tissue. As described above, samples obtained from non-TNBC cancers, metastases, xenografts, single-cell RNA-sequencing, and cell lines, among others, were excluded. We identified six bulk RNA-sequencing studies with suitable TNBC samples, one study with both healthy and TNBC samples, and three additional studies containing adequate healthy control samples. Any samples from these studies that could not be excluded by our standardized set of criteria were included in our analysis to minimize sampling bias. We then manually reviewed the 1355 samples from the 10 studies that met our criteria and reduced them to 196 samples (109 TNBC samples and 87 healthy breast tissue samples; Table 1 and Supplementary File S1).

We then curated and processed the human breast tissue bulk RNA-seq samples to facilitate downstream analyses. Specifically, we used the sequencing reads for each sample as input to the Automated Reproducible MOdular (ARMOR) workflow for preprocessing and a differential analysis of RNA-seq data. In brief, ARMOR performs quality control on samples, trims sequencing reads to remove sequencing adapters and low-quality regions, pseudomaps and quantifies the reads, calculates differentially expressed genes (DEGs), and runs a Gene Ontology enrichment analysis to determine any functions that are significantly affected by TNBC compared to healthy controls.

### 3.2. Differentially Expressed Gene Analysis Reveals Known and New TNBC-Related Transcripts

**Figure 1:**
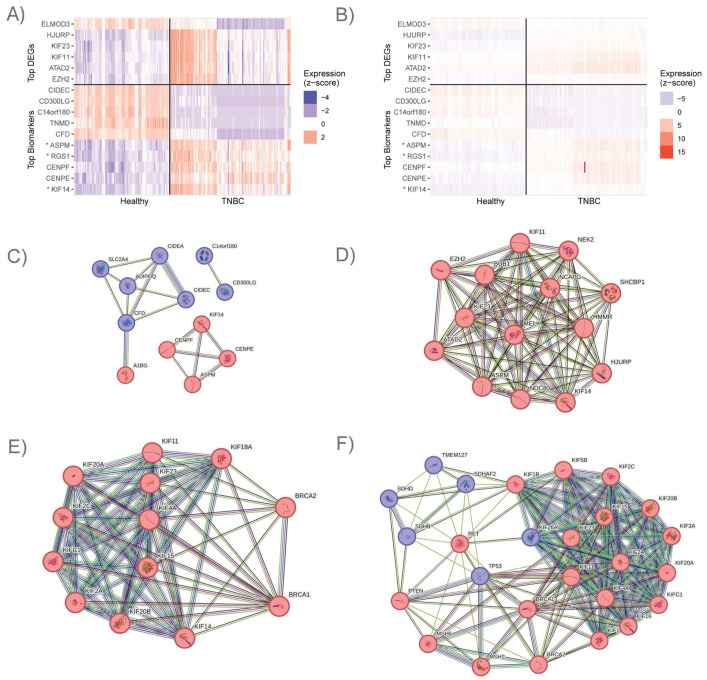
Expression and functionality of top DEGs and mechanistic markers. (**A**) Expression of top-ranking genes that differentiate TNBC from healthy samples after z-score normalization by gene. Gene products with higher expression in TNBC are represented in red, while gene products with lower expression in TNBC are represented in blue. (**B**) Expression of top-ranking genes that differentiate TNBC from healthy samples after z-score normalization by sample. (**C**) Clusters of STRING-db protein–protein interactions among the top 20 mechanistic markers, including 7 downregulated lipid accessibility controllers and 5 upregulated gene products, 4 of which are mitotic. (**D**) Protein–protein interactions between 14 of the top 20 DEGs, which are involved in mitosis and were heavily upregulated. (**E**) Protein–protein interactions between 11 upregulated KIF family proteins and/or BRCA1 and BRCA2. (**F**) Protein–protein interactions between 9 ACMG cancer-related genes, BRCA1 and BRCA2, and 15 KIF family proteins. * ASPM, RGS1, and KIF14 are included in the list of top DEGs and top mechanistic markers. FO082814.1 expression is not shown.

After completing the data preprocessing, we first inspected the ~12,500 differentially expressed genes that significantly differed between TNBC and healthy controls (FDR < 0.05) (Table 2 and Table 3, Figure 1A,B, and Supplementary File S2). The 10 most statistically significant DEGs included KIF14 (Kinesin Family Member 14), ASPM (Assembly Factor for Spindle Microtubules), and RGS1 (Regulator of G Protein Signaling 1), which have previously been connected to TNBC. Furthermore, two novel genes were among the top 10 DEGs: FO082814.1, a Methylenetetrahydrofolate Dehydrogenase (NADP+ dependent) 1-like pseudogene (MTHFD1L), and ELMO Domain Containing 3 (ELMOD3).

We found that the gene product with the most significant *p*-value and a large negative log2 fold change value was FO082814.1, which is annotated as an MTHFD1L-like pseudogene. Interestingly, we observed four MTHFD1L-like pseudogenes to be significantly downregulated in our analysis (Supplementary Table S1). The closest protein-coding homolog to these pseudogenes is MTHFD1L, which is a mitochondrial folate gene [64] that we observed to be slightly but significantly upregulated. To visualize the most significant DEG results, we generated a heatmap by taking the gene read count values for each sample, converting them to a log-base 2 scale, and then applying z-score normalization by gene (Figure 1A) or by sample (Figure 1B). We purposefully selected this normalization approach to minimize the effect of extreme outliers.

### 3.3. Gene Ontology Enrichment Reveals No Significant Terms

We next performed a Gene Ontology (GO) enrichment analysis to determine whether there were any statistically significant functional annotation terms represented by the list of DEGs. Though the Gene Ontology enrichment analysis found 1588 significant (uncorrected *p*-value < 0.05) entities, none of them remained significant after correcting for multiple hypothesis testing (FDR < 0.05; Supplementary File S3). As such, these results were not analyzed further.

### 3.4. Sparse Pathway Modulation Results Precluded Pathway-Based Drug Prediction

Following our analysis of differentially expressed genes and Gene Ontology terms, we next determined whether any intracellular signaling pathways were modulated by TNBC (Table 4 and Supplementary Table S2). We observed that the “PLK1 signaling events” pathway from the NCI pathway database was predicted to be activated, which was expected due to the role that PLK1 plays in metastasis, invasion, and cell division. Interestingly, we also observed that the “integrin signaling pathway” from the BioCarta pathway database was inhibited in the TNBC samples included in our dataset. We attempted to predict therapeutics (and their targets) that could be repurposed for TNBC using the Pathway2Targets algorithm; however, there were no existing targets identified in either of the two significant signaling pathways.

### 3.5. TNBC Samples Do Not Cluster by Predicted TNBC Subtype or Study of Origin

**Figure 2:**
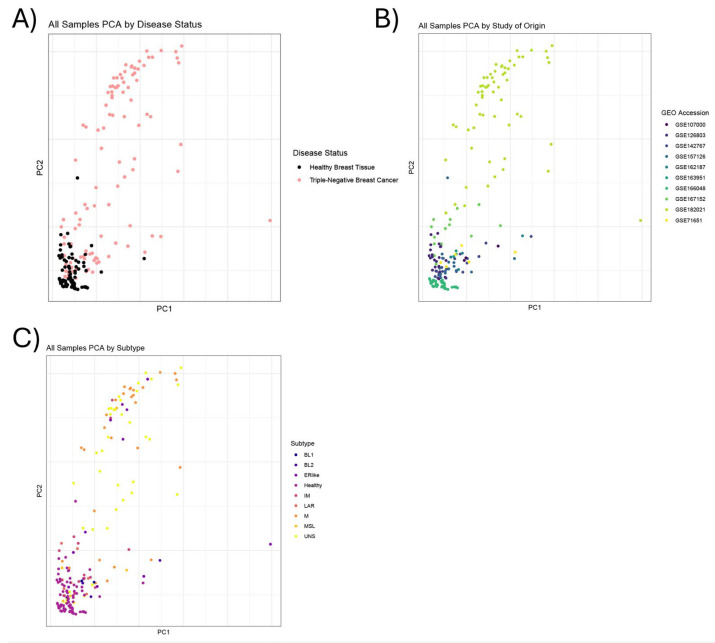
Principal component analysis chart. (**A**) Principal component analysis (PCA) chart showing relatedness of samples, colored by disease status (TNBC vs. Healthy). (**B**) PCA chart with samples colored by study of origin from the Gene Expression Omnibus (GEO). (**C**) Following TNBC subtype prediction, samples (circles) in PCA chart were color-coded according to their predicted subtype, as listed in the subtype key. Subtypes include basal-like 1 (BL1), basal-like 2 (BL2), ER-like (ERlike), healthy (Healthy), immunomodulatory (IM), luminal androgen receptor (LAR), mesenchymal (M), mesenchymal stem-like (MSL), and unspecified (UNS).

Upon the completion of our differential gene expression and pathway enrichment analyses, we wanted to see whether TNBC subtype impacted the pattern of gene expression in our results. Since metadata on TNBC subtype were not available for all samples included in this analysis, the machine learning-based tool TNBCtype (accessed 11 August 2023) was utilized to assign the most likely subtype classification from raw transcript read counts for each sample (Supplementary Files S4 and S5). A subset of 11 samples included in the analysis, which originated from various studies, was predicted by TNBC to be “possible ER-positive samples”. We elected to include these 11 samples in the analysis, and we refer to them as “ERlike” in the subsequent subtype analyses and results since the original studies had classified them as TNBC. Following the prediction of the TNBC subtype for each sample, we performed a principal component analysis (PCA) to visualize the relationship between the predicted subtype and the classified samples (Figure 2; Supplementary Figure S1). Interestingly, the PCA analysis did not cluster the samples by subtype or by study, suggesting that other attributes are more useful in clustering these samples. Unfortunately, the inconsistent availability of additional metadata (e.g., tumor grade and cancer stage) prevented us from identifying the attribute that contributed the most variance to sample clustering. Other than our specification of “ERlike”, the subtypes of TNBC analyzed in this paper include mesenchymal (M), immunomodulatory (IM), luminal androgen receptor (LAR), mesenchymal stem-like (MSL), basal-like 1 (BL1), basal-like 2 (BL2), and unspecified (UNS) [65].

### 3.6. TNMD as a Novel TNBC Mechanistic Marker

Our next step was to predict robust intracellular transcriptional mechanistic markers by using a tree-based machine learning classification algorithm. The justification for this complementary analysis was that the most significant DEGs are commonly indicative of extreme differences in expression between TNBC vs. healthy tissues at the population level. However, many of the top DEGs show little consistency in the level at which they are expressed among individual TNBC samples, making them less accurate as diagnostic markers (Table 5). In an effort to predict high-performing mechanistic markers for TNBC and the common subtypes, we predicted (1) a “universal” set of transcriptional mechanistic markers for all TNBC (vs. healthy control) samples, (2) the usefulness of these “universal” TNBC transcriptional mechanistic markers for each subtype (subtype vs. other subtypes and healthy controls), and (3) the highest-ranking mechanistic markers for each subtype individually (subtype vs. other subtypes and healthy controls).

Our initial mechanistic marker analysis was conducted on the complete set of read counts for all patient samples. Among the top 20 mechanistic marker results, we found several genes known to impact TNBC pathology, including CIDEC (Cell Death Inducing DFFA Like Effector C), CD300LG (CD300 Molecule Like Family Member G), ASPM (Assembly Factor for Spindle Microtubules), CPA1 (Carboxypeptidase A1), CIDEA (Cell Death Inducing DFFA Like Effector A), CENPE (Centromere Protein E), and SLC2A4 (Solute Carrier Family 2 Member 4; Table 5, Supplementary File S6, and Supplementary Tables S3 and S4). We also observed TNMD (Tenomodulin), which is a novel result for TNBC. The TNBC expression consistency of these predicted mechanistic markers as compared to that of the other DEGs is clearly illustrated by visualization in a heatmap (Figure 1A,B). We observed that the top-ranking DEGs tended to have more extreme profiles in some samples, while the predictive mechanistic markers were generally more reliable and consistent in their expression levels.

### 3.7. CIDEC, CD300LG, ASPM, and RGS1 Are 98.9% Effective at Delineating TNBC from Healthy Samples

We then quantified how the best performing “universal” TNBC mechanistic markers performed across individual TNBC subtypes. We consequently performed in-depth predictions of the top five upregulated and the top five downregulated predicted “universal” mechanistic markers to determine their combined and individual predictive accuracy. We ran the mechanistic marker analysis on individual predicted TNBC subtypes vs. healthy samples, according to the subtype classification proposed by Lehmann et al. (Table 6 and Supplementary File S7) [65]. This analysis revealed that one or more of the “universal” TNBC mechanistic markers had good overall performance across many TNBC subtypes, determined by high % ROC-AUC values. Notably, the IM and LAR subtypes both tested at 100% for four of the top mechanistic markers (ASPM, RGS1, CENPF (Centromere Protein F), and KIF11 (Kinesin Family Member 11) for IM; CD300LG, ASPM, CENPF, and KIF14 for LAR). Though these results exceeded expectations, we acknowledge the inherent limitation of this dataset, which is that it does not contain many samples predicted to be from certain subtypes, including BL1, BL2, and MSL. This lack of data makes it difficult to achieve the necessary power to divide the samples into “training” and “test” sets, and it is likely the primary reason for the lower predicted performance of the transcriptional mechanistic markers for these subtypes.

### 3.8. Subtype-Differentiating Mechanistic Markers Include IFNG, AIM2, and FCAMR for IM Subtype

Additionally, we ran mechanistic marker discovery for each predicted subtype to determine which transcripts can be useful in discerning between subtypes and all other samples (subtype vs. healthy with other subtypes; Table 7 and Supplementary File S7). Given the heterogeneity of the TNBC subtypes, we designed this analysis to maximize the specificity and sensitivity while minimizing the number of transcripts that needed to be included. Once again, we found that the subtypes with fewer predicted samples performed less optimally. However, the subtype-specific mechanistic marker analysis appeared to perform well for subtypes with 15 or more samples.

### 3.9. Predicted Mechanistic Markers Contain Potential Repurposable Drug Options

Following the prediction of the subtype-specific mechanistic markers, we ran the Pathway2Targets algorithm on the candidate mechanistic marker genes to discover gene products with known drug–protein interactions [59]. Out of the 37 predicted “universal” TNBC or TNBC subtype-specific mechanistic markers, we found four gene products to have known drugs on the Open Targets database, namely, Interferon Gamma (IFNG), Adrenomedullin (ADM), Phosphodiesterase 3B (PDE3B), and Complement Factor D (CFD). Each of these targets is novel to the treatment of TNBC based on the Open Targets known TNBC drugs (Table 8 and Supplementary Files S8 and S9).

### 3.10. Top DEGs and Mechanistic Markers Include Gene Products Involved in Lipid Utilization, Mitosis, and Intracellular Transport

We observed some interesting patterns when we examined the top DEGs and top mechanistic markers together. Several subnetworks of interacting gene products became evident when we viewed these results using the STRING protein–protein interaction database. Specifically, we observed seven genes that regulate lipid accessibility, transport, and uptake (CD300LG, C14orf180 (Chromosome 14 Open Reading Frame 180), CFD (Complement Factor D), SLC2A4, and ADIPOQ (Adiponectin, C1Q and Collagen Domain Containing)) and lipid droplet surface proteins (CIDEC and CIDEA) to be present in the top 20 mechanistic markers. All of these seven gene products were downregulated in TNBC, and several have other known functional interactions (Figure 1C).

We identified another interesting cluster of gene products that contained a subset of the top 20 DEGs and mechanistic markers, which included eight upregulated mitotic genes. Among these were genes responsible for mitotic spindle/microtubule formation (BUB1 (BUB1 Mitotic Checkpoint Serine/threonine Kinase), ASPM, NDC80 (NDC80 Kinetochore Complex Component), SHCBP1 (SHC Binding and Spindle Associated 1), NEK2 (NIMA Related Kinase 2), and HMMR (Hyaluronan Mediated Motility Receptor)), motor proteins that move the mitotic spindles to accomplish division (KIF11, KIF14, and KIF23 (Kinesin Family Member 23)), and chromosome structure and maintenance (NCAPG (Non-SMC Condensin I Complex Subunit G), ATAD2, HJURP (Holliday Junction Recognition Protein), and EZH2 (Enhancer of Zeste 2 Polycomb Repressive Complex 2 Subunit)). These are known to promote TNBC pathology independently, and they also have known protein–protein interactions with each other (Figure 1D).

Due to the presence of multiple upregulated KIF proteins in the top-ranked DEGs obtained from the TNBC vs. healthy control comparison (30% of the top 10), we wanted to determine whether the KIF family could reveal more about TNBC pathology. Our analysis detected 41 KIF family members in the breast tissue samples included in this study (either healthy or TNBC samples). Among the 41 KIF family gene products that we detected, 25 were significantly upregulated, 7 were significantly downregulated, and 9 lacked significant differential expression. Interestingly, 11 upregulated KIF family proteins directly interact with BRCA1 and BRCA2, 8 of which include wet-lab evidence of protein–protein interactions with BRCA1 or BRCA2 annotated in StringDB (Table 9 and Figure 1E). In addition to the interactions with BRCA1 and BRCA2, we also looked at the interactions between the KIF family proteins and other ACMG cancer-associated genes [52]. We found that 15 KIF family proteins have various connections with 11 of the ACMG cancer-associated genes, including BRCA1 and BRCA2 (Figure 1F). Out of the ACMG cancer-associated genes, six were upregulated in our analysis, five were downregulated, and three were not differentially expressed.

### 3.11. TNBC Top Gene Expression Profiles Validated In Vitro via CellMiner RNA-Seq

Using the CellMiner NCI-60 RNA-Seq data, we validated the expression of the top ten DEGs and mechanistic markers. The CellMiner data cover three TNBC cell lines: MDA-MB-231, HS 578T, and BT-549. To effectively compare the in vitro RNA-seq data and our primary TNBC data, we converted the CellMiner RNA-seq data from units of Fragments Per Kilobase per Million (FPKM) into Transcripts Per Million (TPM) and derived the log2 fold change values using a custom script [66]. Due to the transformed nature of the cell lines, we used the mean TPM from the healthy breast tissue samples in our dataset as the healthy baseline to derive the log2 fold change values for each gene (Table 10). The RNA-seq fold change data from these TNBC cell lines served to confirm the results of our primary patient sample TNBC analysis, with the notable exception of RGS1. We found RGS1 to be aggressively upregulated in our primary TNBC analysis, but this gene product was not detected in the data for any of the three cell lines.

### 3.12. KIF14 and TNMD Gene Products Are Expressed at Expected Levels In Vitro

**Figure 3:**
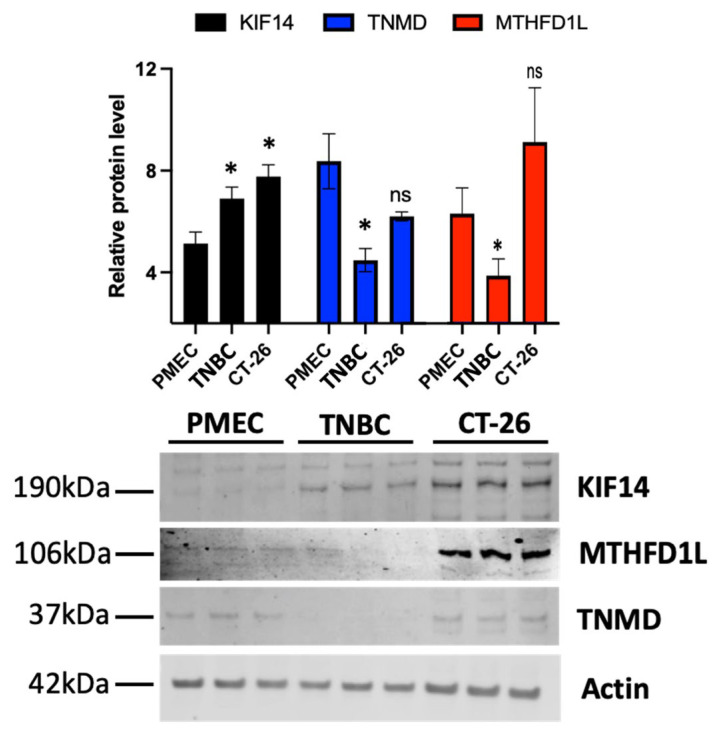
Triple-negative breast cancer shows the dysregulation of KIF14 and TNMD gene products. Immunoblots for KIF14 and TNMD were performed on lysates of primary human breast epithelial cells (PMECs), a triple-negative breast cancer cell line (MDA-MB-231) (TNBC), and a colorectal cancer cell line (CT-26). Student’s *t*-test was used to compare each group against the control primary cells. Asterisks denote the level of significance observed as follows: *, *p*  ≤  0.05; “ns” indicates not significant. Please see Supplementary File S10 for the uncropped Western blots and densitometry information.

We next validated a subset of the results from our bioinformatics-based analysis of the differential gene expression in TNBC using Western blots. To do so, we quantitatively determined the levels of the TNMD, MTHFD1L, and KIF14 proteins in healthy primary mammary epithelial cells (PMECs), a TNBC cell line (MDA-MB-231), and a colon cancer cell line (CT-26). The results for KIF14 and TNMD agreed with our earlier transcript-based results. We purposefully selected these gene products since they were either significantly differentially expressed or predicted mechanistic markers. As found in our transcriptomic analysis, significantly lower TNMD levels were found in the TNBC cells when compared to the primary cells, but there was no significant change in the colon cancer cells (Figure 3 and Supplementary File S10). We also observed that the KIF14 protein levels were significantly increased in the TNBC cells and to an even higher extent in the colorectal cancer cells. Taken together, these data support and validate the observed expression changes in the selected mechanistic markers of TNBC.

As an antibody was not available for our top DEG, the pseudogene FO082814.1, we utilized a polyclonal antibody against MTHFD1L, its closest protein-coding human homolog, to target shared epitopes with any protein produced by the pseudogene (Figure 3 and Supplementary File S10). Our transcriptomic analysis showed a substantial downregulation of FO082814.1 but a slight upregulation of MTHFD1L. Our Western blot showed evidence of a cell type-specific pattern in the levels of MTHFD1L. In particular, the MTHFD1L lanes of the Western blot had significantly lower levels in the TNBC cells when normalized to the PMEC cells, as well as an increase in abundance, which was not significant, in the colorectal cancer cell line.

## 4. Discussion

The purpose of this study was to perform multiple comparative transcriptomic analyses on a large number of TNBC versus healthy breast tissue samples. These analyses focused on predicting differentially expressed genes, pathways, and mechanistic markers relevant to TNBC. Our results highlight two novel DEGs in TNBC, FO082814.1 and ELMOD3, as well as established DEGs, including KIF14, ASPM, KIF11, HJURP, ASPM, EZH2, ATAD2, and RGS1. The presence of genes with known TNBC mechanisms in our results increases our confidence that these bioinformatics results describe genuine biological phenomena. Our in vitro TNBC mRNA expression data from the cell lines BT-549, HS 578T, and MDA-MB-231 reflect expression trends similar to the mRNA expression trends in our patient cohort for the top 10 differentially expressed genes and mechanistic markers (Table 10), corroborating our original findings. Additionally, we identified a novel TNBC mechanistic marker, TNMD, along with other mechanistic markers already known to impact TNBC pathology, including CIDEC, CD300LG, C14orf180, ASPM, RGS1, CFD, CA4, CPA1, CIDEA, CENPF, and CENPE. The presence of both known and novel gene products indicates that the novel highly ranked genes are likely both clinically and biologically relevant. The RNA-seq data from the BT-549, HS 578T, and MDA-MB-231 TNBC cell lines demonstrate gene expression trends similar to our primary tumor in vivo results for the novel genes, further validating our novel results.

Many of our top results have been previously identified and associated with TNBC or other cancer types, which at least partially corroborates our methods and results. Interestingly, two inflammation-related heme proteins previously associated with poor TNBC outcomes, Indoleamine 2,3-Dioxygenase 1 (IDO1) and Tryptophan 2,3-Dioxygenase (TDO2) [67,68,69,70,71], were also found to be upregulated in the current study. However, the associations between TNBC and FO082814.1 and ELMOD3 are novel to this analysis. FO082814.1, the most significant DEG, has not previously been researched in TNBC or otherwise. The closest related gene to FO082814.1 is MTHFD1L, a mitochondrial dehydrogenase, which was upregulated in this study. The presence of four significantly downregulated MTHFD1L-like pseudogenes, including FO082814.1, in our analysis suggests a potential common regulatory role for them in TNBC. It is also possible that the calculated differential expression of this pseudogene was at least partially due to the inability of the Salmon pseudomapping to accurately differentiate between the gene and the pseudogene. Additional future work will be required to better characterize any role that MTHFD1L and/or FO082814.1 plays in TNBC. Our analysis also identified the ELMO Domain Containing 3 (ELMOD3) gene to be downregulated in TNBC. ELMOD3 has been reported in past breast cancer studies [72,73], which neither disagree with nor invalidate our findings. In fact, this is the first study that associates transcriptional changes in ELMOD3 with human TNBC.

KIF family members, used for cell motility and spindle separation in mitosis, appear frequently in the top 10 significant DEGs. The upregulation of KIF23 [74,75,76], KIF11 [77,78], and KIF14 [79] in TNBC has previously been reported. Currently, KIF23 [74,75] and KIF11 [78] are under investigation as TNBC drug targets; KIF11 inhibition was previously shown to stall the tumor growth of breast cancer xenografts [77], while KIF23 drove TNBC migration, proliferation, and the metastasis-promoting epithelial–mesenchymal transition in vitro [74,75]. KIF14 is involved in proliferation, and an elevated KIF14 expression is associated with chemotherapy-resistant TNBC [80], making it an ideal target for refractory cases. To the best of our knowledge, no previous work has been conducted on KIF14 as a drug target in TNBC. Among known membrane-associated cancer mechanisms, KIF14 co-localizes at the cell membrane with AKT and activates AKT by phosphorylation in TNBC, thus starting the signal cascade for the PI3K/AKT pathway [80]. In lung adenocarcinoma, KIF11 recruits CDH11 to the cell membrane and is known to bind to CDH11; CDH11 is a known outer membrane protein and contributes to cell metastasis [81]. High-KIF14-expressing cells have recently been shown to help form torpedo-like invasive complexes at the tumor–healthy cell interface, with cells expressing the most KIF14 being associated with high metastatic potential [82]. These data suggest that some KIF proteins may have regular interactions with the cell membrane. Additional characterization of the KIF proteins is required to determine their specificity as protein or cell surface markers in TNBC.

Following the determination of TNBC mechanistic markers using machine learning, we noticed that several of the most highly ranked results were upregulated. Abnormal Spindle Microtubule Assembly (ASPM) was the best-ranking upregulated mechanistic marker in our dataset. ASPM has been investigated as a TNBC drug target [83]. The knockdown of ASPM inhibited TNBC growth and killed tumor cells in vitro [84]. Regulator of G Protein Signaling 1 (RGS1) was our second-ranking upregulated mechanistic marker and among the top 10 DEGs. RGS1 is a germinal center chemokine regulator for B-cells, and it is expressed at higher levels in tumor-infiltrating B-cells than in circulating B-cells [85]. Both CD4+ and CD8+ T-cells exhibit reduced trafficking to and survival in tumors with an elevated RGS1 expression [86]. Centromere Protein F (CENPF) and Centromere Protein E (CENPE) were the next highest-ranked predicted TNBC mechanistic markers upregulated in TNBC. CENPE has previously been drug-targeted in vitro in TNBC with substantial success [87]. CENPF is highly expressed in TNBC, and its high expression is correlated with low survival. Wang et al. showed that knocking down CENPF resulted in an increased efficacy of the chemotherapeutic agent adriamycin [88].

Among the downregulated mechanistic markers described in this study, several have promising relevance based on prior work. Cell Death Inducing DFFA Like Effector C (CIDEC) was downregulated by TNBC and was the top-ranking predictive mechanistic marker. CIDEC has previously been shown to be downregulated in breast cancer but not specifically in TNBC [89,90]. CIDEC is negatively regulated by estrogen and is involved in apoptosis in breast tissue [90], perhaps explaining the specific downregulation in breast tissue despite reports of CIDEC upregulation in cancers of other tissue types [89]. CD300 Molecule Like Family Member G (CD300LG), the second-ranked downregulated TNBC mechanistic marker in our analysis, was previously reported as being downregulated in breast tumor tissue [91,92]. CD300LG is critical to lymphocyte diapedesis and has very low expression in immunoprivileged locations in the body (the brain, testes, uterus, and gut) to keep lymphocytes out [93]. Its suspected mechanism in cancer is to help tumors avoid the immune system by not allowing lymphocytes to reach the tumor. Additionally, we found a promising mechanistic marker that is novel for TNBC. Tenomodulin (TNMD) was the sixth-highest predicted transcriptional marker and was downregulated in primary TNBC and all three cell lines wherein TNMD mRNA expression was tested (BT-549, HS 578T, and MDA-MB-231). Tenomodulin (TNMD) is a transmembrane glycoprotein and is highly expressed in both developing and mature tendons. Although its function is unknown, it has been reported to have anti-angiogenic properties [94]. Since TNMD was downregulated by TNBC, it could also potentially play a role in inhibiting angiogenesis in breast tissue. Since TNMD has little to no expression in MDA-MB-231 TNBC cells, it may be a good negative surface marker. Alternatively, it could be a reliable marker on the surface of non-TNBC breast cells, which could be useful for tumor-delineating surgical stains.

An interesting trend in the high-ranking results was the presence of downregulated lipid droplet genes. Lipid utilization has previously been noted as an important TNBC feature by a bioinformatics hub gene enrichment analysis [95]. The top 20 mechanistic markers that we found included seven genes that regulate lipid accessibility and metabolism, namely, CIDEC, CD300LG, C14orf180, CFD, CIDEA, ADIPOQ, and SLC2A4, all of which were substantially downregulated in TNBC. Though CIDEC and CD300LG have been shown to be downregulated in other breast cancers, they have not previously been identified in TNBC [89,90,91,92]. CD300LG is a protein expressed in the membrane of myocytes and adipocytes that controls intracellular lipid content, is associated with metabolism and glucose uptake, and contributes to cell motility [96]. SLC2A4 and C14orf180 downregulation is linked to poor breast cancer outcomes, with lower expression associated with lower survival [97,98]. CIDEC loss of function in the lung deregulates lipase activity and produces lipid-laden neutrophils, which feed their lipid stores to metastasizing TNBC cells via micropinocytosis, stabilizing metastatic TNBC grafts to the lung [99]. This lipid-laden neutrophil phenotype may also be possible in breast tissue with the downregulation of CIDEC function, though no characterization in TNBC has taken place. CIDEC and CIDEA localize to the surface of lipid droplets inside of normal cells [100]. In lean individuals, and presumably also energy-starved environments, such as the tumor microenvironment, ADIPOQ encourages lipid storage [101]. Additionally, the presence of C14orf18, CFD, and CIDEA enhances lipid storage, and CIDEA prevents lipid use by sequestering the lipids to the lipid droplets [102,103,104]. The downregulation of these genes deregulates and destabilizes lipid droplets, making them more accessible for lipid metabolism, though such characterizations in TNBC are lacking. Glucose uptake, lipid utilization, and insulin creation and sensitivity are all affected when lipid utilization is deregulated [96,102]. These known underlying functions of our downregulated lipid-associated mechanistic markers could provide direction for understanding why patient outcomes are affected by these genes.

Of our four mechanistic markers that are targets of existing therapeutics that could be repurposed for TNBC, three are already in experimentation in vitro, and one, CFD, is novel. There is growing evidence suggesting that complement proteins are not always secreted and sometimes have roles beyond innate immunity via cell lysis and chemokine signaling [105]. Though little is known about CFD in terms of its involvement in TNBC, higher CFD expression is a known risk factor for TNBC prognosis [106]. PDE3B is a potent TNBC-promoting agent, and drug antagonism of PDE3B in TNBC can slow tumor growth and prevent metastasis to the lungs [107]. IFNG has been previously noted as a potential TNBC target and has a complex relationship with TNBC; while it encourages potent antitumor activity via CD-8+ T-cell activation and other immunological mechanisms, increased IFNG levels can also induce PDL1 expression in some TNBC tumors, which presents a hazard to the patient due to the CD-8+ T-cell exhaustion caused by the PD1-PDL1 checkpoint interaction [108]. The pharmaceutical agent triptolide can reverse IFNG-inducible PDL1 expression in TNBC, improving the safety of using IFNG and IFNG agonists for TNBC treatment [108]. Higher ADM expression has similarly been identified as a positive prognostic marker for TNBC, and ADM is suspected to decrease the potential for metastasis by negatively regulating the epithelial–mesenchymal transition [109]. These data recommend CFD, PDE3B, IFNG, and ADM as agents for further study as TNBC targets. Additional research is needed to determine the efficacy of CFD as a TNBC target, and the presence of three targets with already known anti-TNBC efficacy increases our confidence in the utility of our mechanistic marker results.

This study has several limitations that may affect the general relevance of the findings. Firstly, we purposefully selected RNA-seq data from only TNBC patients and excluded all other types of breast cancer. Our analysis was therefore specific to TNBC, and we did not directly compare it with other types of breast cancer. Although we experimentally validated a subset of our findings, which increases our confidence in them, additional future experiments will be needed to similarly validate other important findings in this study. Secondly, some studies focus on a well-defined patient population to maximize the accuracy of their results at the expense of generalized relevance. Our study incorporated a large number of diverse samples in an effort to improve general relevance at the expense of accuracy in small subpopulations of human patients. Despite our efforts to include the maximum number of TNBC samples in GEO, some TNBC subtypes were less represented in our dataset. We assumed that the clusters in the PCA plot were likely grouped by tumor stage; however, we were unable to prove this hypothesis given the lack of metadata for this attribute. Though we did our best to analyze the TNBC subtypes based on the included samples, a more thorough treatment of TNBC subtypes was beyond the scope of this study. We show reliable overall mechanistic markers and good directions for subtype-specific mechanistic markers that can be utilized and confirmed by physicians who have more access to samples. Thirdly, though we included the subtype-specific mechanistic marker prediction results for all subtypes, the low number of samples for some of the subtypes in our dataset precluded clear results for these categories. We do not feel that this limitation invalidates the other results, as the overall mechanistic markers performed very well for the predicted subtypes with six or more samples. Our study was specific to TNBC, so any comparison with other breast cancer types and subtypes was deemed out of scope. Lastly, the de-identified metadata that were available did not include cancer stage. As such, we expect that our analysis was skewed away from earlier stages of cancer, which likely have an effect on our mechanistic and mechanistic marker findings across patients in each stage of TNBC.

In this study, the genes that we found to be significant in a 200-sample patient cohort were validated by RNA-seq and Western blot expression studies in vitro. This tri-fold verification gives us confidence that our results consist of real biological phenomena and supports the future study of CIDEC and TNMD as mechanistic markers, FO082814.1 as a novel TNBC-associated pseudogene, and KIF14 as a therapeutic target. Future steps include upregulation and downregulation studies in the TNBC cell lines MDA-MB-231 and MDA-MB-453 using methods such as CRISPRa and CRISPRi to determine the effects of the top gene results on tumor migration and proliferation in vitro. We anticipate that this work will lead to promising new avenues for patient differentiation and personalized TNBC treatment as these tri-validated results are further validated by additional wet-lab assays.

## 5. Conclusions

Our secondary analysis of TNBC vs. healthy human breast tissue consisted of nearly 200 publicly available samples from 10 studies. Our analyses revealed characterized and novel gene products that augment our understanding of the mechanisms of TNBC as a whole and its individual subtypes. We subsequently validated our clinical gene expression findings by analyzing the mRNA expression trends in the TNBC cell lines BT-549, HS 578T, and MDA-MB-231. These in vitro gene expression results corroborated our findings in the in vivo patient dataset. Several predicted mechanistic markers for TNBC subtypes were found to be drug-targetable based on known drug–protein interactions. The robustness of the TNBC mechanistic markers from breast tissue could be used to develop improved diagnostics that can lead to earlier and/or a more accurate detection of TNBC patients in the future.

## Figures and Tables

**Table 1:** The 10 publicly available sequencing projects included in this study.

Sample Phenotype	Single-End or Paired-End Reads	GEO Accession Number	Relevant Samples
TNBC	Paired End	GSE163951 (Unpublished)	3
TNBC	Single End	GSE182021 [19]	61
TNBC	Paired End	GSE142767 [20]	21
TNBC	Paired End	GSE107000 [21]	3
TNBC *	Paired End	GSE71651 [22,23,24,25,26]	3
TNBC	Single End	GSE167152 [27]	14
TNBC	Paired End	GSE162187 [28,29]	4
Healthy Breast Tissue *	Paired End	GSE71651 [22,23,24,25,26]	3
Healthy Breast Tissue	Single End	GSE126803 (Unpublished)	23
Healthy Breast Tissue	Single End	GSE157126 [30]	16
Healthy Breast Tissue	Paired End	GSE166048 [31]	45

* GSE71651 contains both TNBC and healthy breast tissue samples and appears in the table twice for clarity.

**Table 2:** Most significant differentially expressed genes (ascending rank by FDR) in TNBC.

Gene Symbol	Gene Name According to HUGO Gene Nomenclature Committee	Log_2_ Fold Change	False Discovery Rate (FDR) *p*-Value
FO082814.1	Methylenetetrahydrofolate Dehydrogenase (NADP+ dependent) 1 Like (MTHFD1L) Pseudogene	−8.05	5.82 × 10^−57^
KIF14	Kinesin Family Member 14	5.6	8.70 × 10^−56^
ELMOD3	ELMO Domain Containing 3	−3.59	4.20 × 10^−55^
HJURP	Holliday Junction Recognition Protein	4.55	1.62 × 10^−53^
KIF23	Kinesin Family Member 23	3.92	2.99 × 10^−53^
KIF11	Kinesin Family Member 11	3.5	3.62 × 10^−53^
ASPM	Abnormal Spindle Microtubule Assembly	6.53	4.37 × 10^−53^
ATAD2	ATPase Family AAA Domain Containing 2	3.41	4.95 × 10^−53^
EZH2	Enhancer of Zeste 2 Polycomb Repressive Complex 2 Subunit	3.55	5.05 × 10^−52^

**Table 3:** Highest ranking gene products by descending log_2_ fold change.

Gene Symbol	Gene Name According to HUGO Gene Nomenclature Committee	Log_2_ Fold Change	False Discovery Rate (FDR) *p*-Value
IBSP	Integrin Binding Sialoprotein	10.4	3.83 × 10^−36^
MMP1	Matrix Metallopeptidase 1	9.69	7.97 × 10^−48^
HIST1H2BB	Histone Cluster 1 H2B Family Member B	9.08	1.68 × 10^−31^
CASP14	Caspase 14	9.03	9.78 × 10^−27^
HIST1H3B	Histone Cluster 1 H3 Family Member B	8.59	2.05 × 10^−33^
HIST1H1B	Histone Cluster 1 H1 Family Member B	8.48	1.47 × 10^−31^
S100A7	S100 Calcium Binding Protein A7	8.4	1.60 × 10^−30^
RIMBP3C	RIMS Binding Protein 3C	8.38	4.75 × 10^−27^
COL10A1	Collagen Type X Alpha 1 Chain	8.31	1.41 × 10^−44^

**Table 4:** Differentially modulated pathways in TNBC.

	Name	pSize *	NDE	Direction of Modulation	Bonferroni-Adjusted *p*-Value	Pathway Database
1	PLK1 signaling events	44	42	Activated	8.62 × 10^−6^	NCI
2	Integrin signaling pathway	37	28	Inhibited	6.98 × 10^−3^	BioCarta

* pSize: number of entities in the pathway, NDE: number of differentially expressed genes in the pathway.

**Table 5:** Summarized information for top five up- and down-regulated mechanistic markers in TNBC.

Rank	Gene Symbol	Gain	Disease Status	Mean (Read Counts)	Standard Deviation (Read Counts)	Median (Read Counts)	Log_2_ Fold Change	False Discovery Rate
1	CIDEC	0.26	TNBC	8.28	31.06	1	−7.9	3.73 × 10^−45^
			Healthy	1687.83	1495.58	1363		
2	CD300LG	0.12	TNBC	9.94	26.56	0	−6.49	1.76 × 10^−48^
			Healthy	506.45	370.17	461		
3	C14orf180	0.09	TNBC	3.19	10.39	0	−7.88	6.19 × 10^−46^
			Healthy	668.37	705.87	331		
4	ASPM	0.09	TNBC	782.43	1327.45	392	6.53	4.37 × 10^−53^
			Healthy	15.72	17.43	10		
5	RGS1	0.06	TNBC	395.07	734.93	154	6.53	5.05 × 10^−52^
			Healthy	8.57	11.18	5		
6	TNMD	0.05	TNBC	1.06	2.61	0	−5.76	1.58 × 10^−36^
			Healthy	80.77	95.61	47		
7	CFD	0.05	TNBC	43.26	112.94	2.5	−6.53	5.59 × 10^−34^
			Healthy	1078.11	1282.38	683.5		
14	CENPF	0.02	TNBC	662.05	795.07	409	5.52	3.46 × 10^−41^
			Healthy	30.74	26.31	26		
15	CENPE	0.01	TNBC	265.10	256.41	195	6.66	2.98 × 10^−43^
			Healthy	12.25	15.59	7		
18	KIF14	0.01	TNBC	127.28	167.56	63	5.6	8.70 × 10^−56^
			Healthy	4.52	4.29	3		

**Table 6:** Predictive capability of mechanistic markers by TNBC subtype (% ROC-AUC).

	* All TNBC	UNS	M	IM	ERlike	LAR	MSL	BL1	BL2
Number of Samples	109	37	29	15	11	6	5	3	3
**CIDEC, CD300LG, ASPM, RGS1 (top 2 upregulated and top 2 downregulated)**	98.9	99.3	100.0	100.0	83.3	100.0	100.0	50.0	50.0
**CIDEC, CD300LG (top 2 downregulated)**	97.1	99.3	100.0	90.9	88.2	100.0	99.3	50.0	50.0
**ASPM, RGS1 (top 2 upregulated)**	96.1	98.3	97.8	100.0	83.3	100.0	50.0	50.0	50.0
**CIDEC**	97.1	99.3	99.3	87.7	88.2	99.3	99.3	50.0	50.0
**CD300LG**	94.4	98.3	100.0	91.6	77.8	100.0	90.0	50.0	50.0
**C14orf180**	96.8	99.3	97.1	83.2	87.5	82.6	89.3	50.0	50.0
**ASPM**	95.8	96.0	97.8	100.0	81.9	100.0	50.0	50.0	50.0
**RGS1**	94.8	96.0	93.5	100.0	82.6	90.9	50.0	50.0	50.0
**TNMD**	94.8	98.6	94.2	94.7	81.2	90.9	89.3	50.0	50.0
**CFD**	95.3	96.0	97.1	91.6	88.2	99.3	50.0	50.0	50.0
**CENPF**	91.4	96.0	94.2	100.0	76.3	100.0	50.0	50.0	50.0
**CENPE**	92.5	92.9	92.8	95.4	81.9	99.3	50.0	50.0	50.0
**KIF14**	92.1	94.3	94.2	100.0	81.9	100.0	50.0	50.0	50.0

* TNBC: triple-negative breast cancer, UNS: unspecified, M: mesenchymal, IM: immunomodulatory, ERlike: estrogen receptor-like, LAR: luminal androgen receptor, MSL: mesenchymal stem-like, BL1: basal-like 1, BL2: basal-like 2.

**Table 7:** Mechanistic markers specific to each TNBC subtype.

Subtype	Number of Subtype Samples	Top 3 Subtype-Differentiating Biomarkers	% ROC-AUC (Combined Accuracy of Top 3 Subtype-Specific Biomarkers)
* All TNBC	109	CIDEC, CD300LG, C14orf180	97.1
UNS	37	FGF14, ADM, SH3PXD2A	88.5
M	29	GBP4, ARRB1, PDE3B	82.2
IM	15	IFNG, AIM2, FCAMR	91.3
ERlike	11	ASCL1, IQCJ, CDKN2A	70.0
LAR	6	ABCA12, ABCC12, AADAT	70.0
MSL	5	CH25H, ABCA2, AATK	70.0
BL1	3	ABHD13, ANKHD1, AGTPBP1	50.0
BL2	3	ACP6, AAGAB, AAAS	50.0

* TNBC: triple-negative breast cancer, UNS: unspecified, M: mesenchymal, IM: immunomodulatory, ERlike: estrogen receptor-like, LAR: luminal androgen receptor, MSL: mesenchymal stem-like, BL1: basal-like 1, BL2: basal-like 2.

**Table 8:** Mechanistic marker gene products with potential for drug repurposing.

Target Symbol	Target Name	Relevant TNBC Subtype	Number of Unique Drugs	Number of Approved/Phase 4 Drugs	Number of Phase 3 Drugs	Number of Phase 2 Drugs	Number of Phase 1 Drugs
IFNG	Interferon Gamma	IM	3	1	0	0	0
ADM	Adrenomedullin	UNS	1	0	0	1	0
PDE3B	Phosphodiesterase 3B	M	11	0	1	0	0
CFD	Complement Factor D	All TNBC	2	0	1	0	0

**Table 9:** KIF genes that have direct protein–protein interactions with BRCA1 or BRCA2.

Gene Symbol	Log2 FC	FDR *p*-Value
KIF23	3.92	2.99 × 10^−53^
KIF11	3.50	3.62 × 10^−53^
KIF14	5.60	8.70 × 10^−56^
KIF2C	4.30	1.58 × 10^−44^
KIF18A	4.52	3.70 × 10^−46^
KIF4A	5.02	2.47 × 10^−43^
KIF15	4.91	2.29 × 10^−43^
KIF20A	4.26	3.76 × 10^−34^
KIF20B	2.93	5.86 × 10^−36^
KIF2A	2.03	1.12 × 10^−26^

**Table 10:** TNBC cell line RNA-seq fold changes support top 10 DEGs and mechanistic markers.

Gene Name	Average Primary TNBC (Log_2_FC)	BT-549 (Log_2_FC)	HS 578T (Log_2_FC)	MDA-MB-231 (Log_2_FC)
MTHFD1L	1.47	2.81	2.29	2.1
KIF14	5.6	6.87	6.67	7.49
ELMOD3	−3.59	−3.54	−3.36	−6.01
HJURP	4.55	6	6.31	5.94
KIF23	3.92	3.43	4.34	5.98
KIF11	3.5	3.74	3.47	5.27
ASPM	6.53	6.38	6.52	8.44
ATAD2	3.41	2.48	2.66	2.85
EZH2	3.55	3.58	2.68	4.76
RGS1	6.53	ND *	ND *	ND *
CIDEC	−7.9	−6.09	−5.92	ND *
CD300LG	−6.49	ND *	−5.56	ND *
C14orf180	−7.88	ND *	−4.81	ND *
TNMD	−5.76	ND *	ND *	ND *
CFD	−6.53	−3.64	−7.93	−8.36
CENPF	5.52	3.24	3.72	5.17
CENPE	6.66	3.38	3.67	6.65

* ND: not detected.

## Data Availability

The datasets analyzed during the current study are available in the Gene Expression Omnibus (GEO) repository. All GEO accession numbers for the included sequencing projects can be found in Table 1, while individual SRA identifiers for the included samples can be viewed in Supplementary File S1. All custom scripts are cited in the text and are accessible at https://zenodo.org/doi/10.5281/zenodo.12752864 (accessed on 1 October 2024). Supplementary File S7 is available for download at https://doi.org/10.5281/zenodo.10251085 (accessed on 1 October 2024). All other data generated or analyzed during this study are included in this published article and its Supplementary Materials files.

## Supplementary Materials

The following supporting information can be downloaded at: https://www.mdpi.com/article/10.3390/cancers16193379/s1, Figure S1: Overview of studies and samples included in analysis; Table S1: Four Chromosome-9 MTHFD1L-like pseudogenes have significant differential expression in TNBC; Table S2: Detailed SPIA pathway modulation results; Table S3: TNBCtype subtype prediction results; Table S4: Tree-based mechanistic marker prediction summary statistics for all 196 samples; File S1: Included sample experimental metadata; File S2: Complete edgeR differentially expressed gene results table; File S3: Camera Gene Ontology results table; File S4: Raw transcript read count matrix used to predict mechanistic markers and visualize mechanistic marker and differentially expressed gene results; File S5: TNBCtype subtype prediction results; File S6: Tree-based mechanistic markers ranked by ability to correctly predict healthy vs. TNBC for all 196 samples; File S7: G-zipped folder of tree-based mechanistic marker output files corresponding to Table 6 and Table 7; File S8: TNBC mechanistic markers with known drugs and Pathway2Targets complete output; File S9: Specific drug information for mechanistic marker targets; File S10: Uncropped Western blot gel images.
