# Supplementary material for: Secondary Transcriptomic Analysis of Triple-Negative Breast Cancer Reveals Reliable Universal and Subtype-Specific Mechanistic Markers
Source: Cancers (Basel). 2024 Oct 2;16(19):3379. doi: 10.3390/cancers16193379 (PMC11476281; doi:10.3390/cancers16193379)
Supplement: Supplementary file 1 [file cancers-16-03379-s001.zip › Supplementary_File_S0_Tables_and_Figures.pdf]

Supplementary Material

1 Supplementary Data

Supplementary File S1: Included Sample Experimental Metadata

Supplementary File S2: Complete EdgeR Differentially Expressed Gene Results Table

Supplementary File S3: Camera Gene Ontology Results Table

Supplementary File S4: Raw Transcript Read Count Matrix Used to Predict Mechanistic Markers and Visualize Mechanistic Marker and Differentially Expressed Gene Results

Supplementary File S5: TNBCtype Subtype Prediction Results

Supplementary File S6: Tree-Based Mechanistic Markers Ranked by Ability to Correctly Predict Healthy vs. TNBC for all 196 Samples

Supplementary File S7: G-zipped Folder of Tree-based Mechanistic Marker Output Files Corresponding to Tables 4-5

Supplementary File S8: TNBC Mechanistic Markers with Known Drugs Pathway2Targets Complete Output

Supplementary File S9: Specific Drug Information for Mechanistic Marker Targets Pathway2Targets Output

Supplementary File S10: Uncropped Western Blot Gel Images

2 Supplementary Figures and Tables

2.1 Supplementary Tables

Supplementary Table S1: Four Chromosome-9 MTHFD1L-like Pseudogenes have Significant Differential Expression in TNBC

* MTHFD1L: Methylenetetrahydrofolate Dehydrogenase (NADP+ dependent) 1 like, FDR: False Discovery Rate.

**Gene Name**	**Chromosome**	**Log_2 Fold Change**	**FDR-Corrected p-value**
* MTHFD1L	6	1.47	1.07×10^{-18}
FO082814.1	9	-8.05	5.82×10^{-57}
FP325317.2	9	-5.93	3.77×10^{-37}
AL591379.1	9	-2.73	6.75×10^{-19}
AL445665.1	9	-2.25	1.21×10^{-07}

Supplementary Table S2: Detailed SPIA Pathway Modulation Results

* pSize: pathway size (number of genes), NDE: Number of Differentially Expressed genes in pathway, pNDE: probability to observe at least NDE genes on the pathway using a hypergeometric model, tA: total perturbation Accumulation in the pathway (also indicates modulation directionality), pPERT: the probability to observe a total accumulation more extreme than tA only by chance, pG: the p-value obtained by combining pNDE and pPERT, pGFdr: False Discovery Rate adjusted global p-value, pGFWER: Bonferroni adjusted global p-value, SourceDB: Source Database of pathway, PLK1: Polo-Like Kinase 1, NCI: National Cancer Institute.

	Name	* pSize	NDE	pNDE	tA	pPERT	pG	pGFdr	pGFWER	Status	SourceDB
1	integrin signaling pathway	37	28	0.591409	-58.1885	5.00E-06	4.06E-05	0.006984	0.006984	Inhibited	BioCarta
2	PLK1 signaling events	44	42	0.00054	307.6052	5.00E-06	5.60E-08	8.62E-06	8.62E-06	Activated	NCI

Supplementary Table S3: Tree-based Mechanistic Marker Prediction Results for All 196 Samples

Prediction	Reference	
0	1	
0	70	2
1	0	85

Supplementary Table S4: Tree-based Mechanistic Marker Prediction Summary Statistics for All 196 Samples

* NIR: No Information Rate.

Label	Value
Accuracy	0.987261146
95% Confidence Interval	0.954742240094415 - 0.998453525367956
No Information Rate	0.554140127
* P-Value [Accuracy > NIR]	4.51E-37
Kappa	0.974291796
Mcnemar's Test P-Value	0.479500122
Sensitivity	1
Specificity	0.977011494
Positive Predictive Value	0.972222222

Negative Predictive Value	1
Precision	0.972222222
Recall	1
F1 (harmonic mean of precision and recall)	0.985915493
Prevalence	0.445859873
Detection Rate	0.445859873
Detection Prevalence	0.458598726
Balanced Accuracy	0.988505747
'Positive' Class	0

2.2 Supplementary Figures

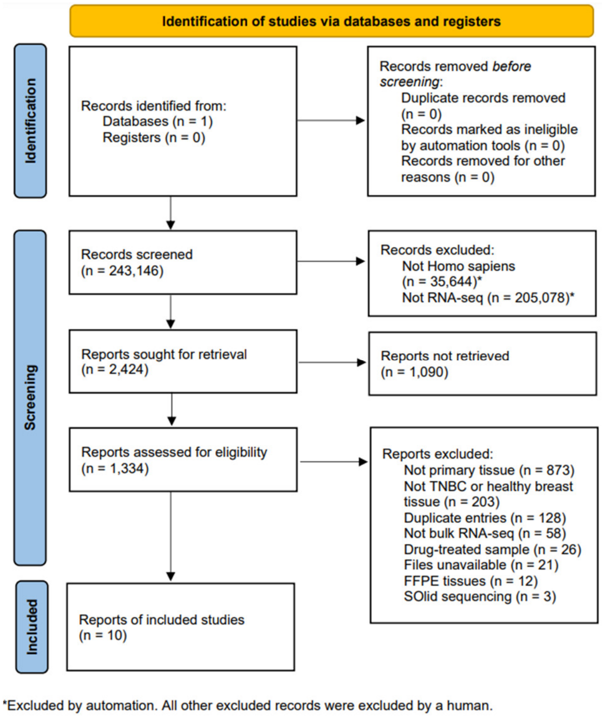

Supplementary Figure S1: Overview of Studies and Samples Included in Analysis. The PRISMA flow diagram for transparent reporting of joint secondary analyses. This chart indicates the search procedures we used to obtain and define our dataset.
