# Supplementary material for: Secondary Transcriptomic Analysis of Triple-Negative Breast Cancer Reveals Reliable Universal and Subtype-Specific Mechanistic Markers
Source: Cancers (Basel). 2024 Oct 2;16(19):3379. doi: 10.3390/cancers16193379 (PMC11476281; doi:10.3390/cancers16193379)
Supplement: Supplementary file 1 [file cancers-16-03379-s001.zip › Supplementary_File_S10_JMN_TNBC_Uncropped_Gel_Images_and_Densitometry.pptx]

## Slide 1
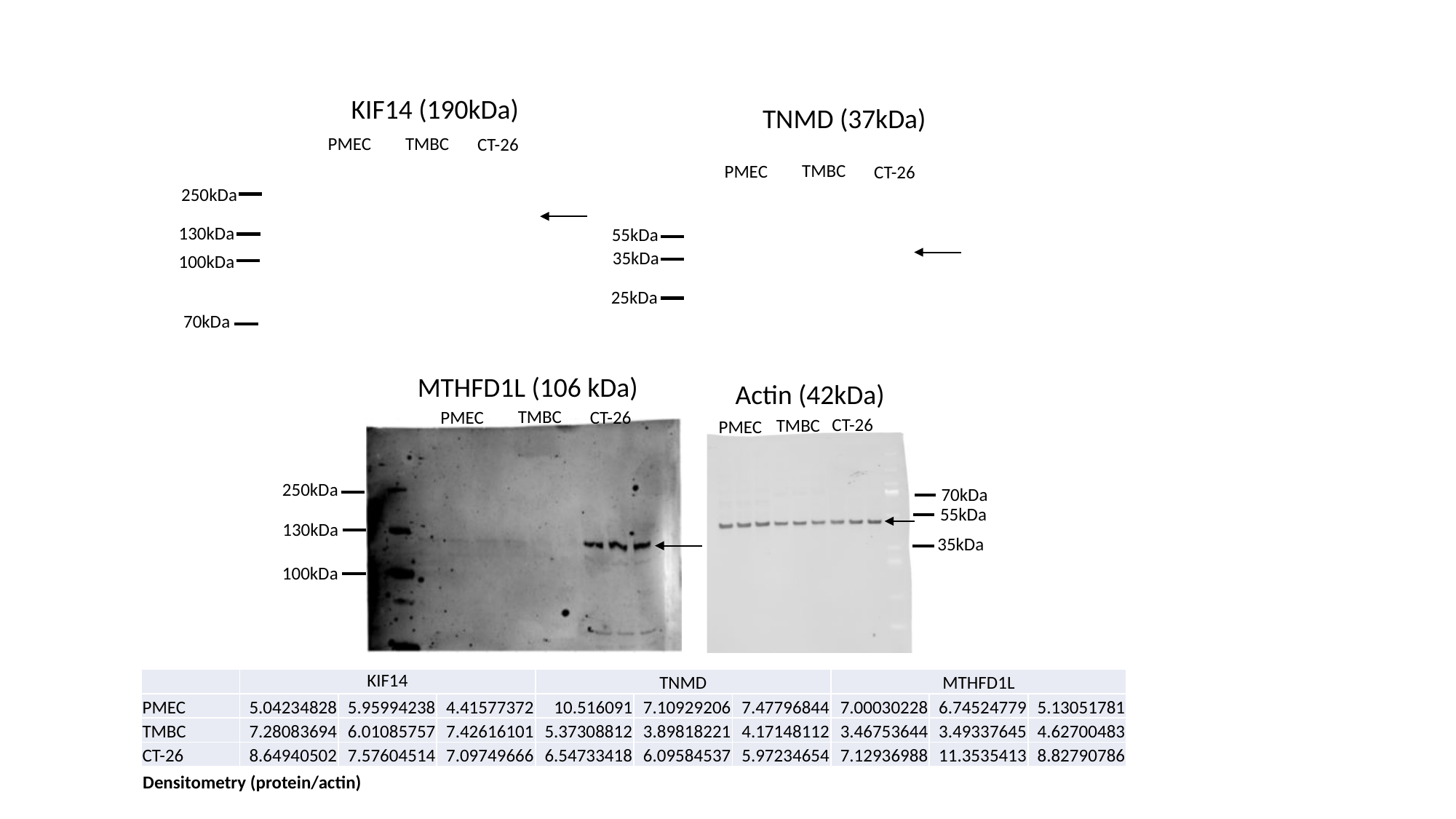

KIF14 (190kDa)
TNMD (37kDa)
TMBC
PMEC
CT-26
TMBC
PMEC
CT-26
250kDa
130kDa
55kDa
35kDa
100kDa
25kDa
70kDa
MTHFD1L (106 kDa)
Actin (42kDa)
TMBC
PMEC
CT-26
CT-26
TMBC
PMEC
250kDa
70kDa
55kDa
130kDa
35kDa
100kDa
| | KIF14 | | | TNMD | | | MTHFD1L | | |
| --- | --- | --- | --- | --- | --- | --- | --- | --- | --- |
| PMEC | 5.04234828 | 5.95994238 | 4.41577372 | 10.516091 | 7.10929206 | 7.47796844 | 7.00030228 | 6.74524779 | 5.13051781 |
| TMBC | 7.28083694 | 6.01085757 | 7.42616101 | 5.37308812 | 3.89818221 | 4.17148112 | 3.46753644 | 3.49337645 | 4.62700483 |
| CT-26 | 8.64940502 | 7.57604514 | 7.09749666 | 6.54733418 | 6.09584537 | 5.97234654 | 7.12936988 | 11.3535413 | 8.82790786 |
Densitometry (protein/actin)
