# Supplementary material for: Secondary Transcriptomic Analysis of Triple-Negative Breast Cancer Reveals Reliable Universal and Subtype-Specific Mechanistic Markers
Source: Cancers (Basel). 2024 Oct 2;16(19):3379. doi: 10.3390/cancers16193379 (PMC11476281; doi:10.3390/cancers16193379)
Supplement: Supplementary file 1 [file cancers-16-03379-s001.zip › Supplementary_File_S7_Output_File_Tables_4-5.pdf]

Supplementary File S7: G-zipped Folder of Tree-based Mechanistic Marker Output Files Corresponding to Tables 4-5 (Supplementary_File_S10_Output_File_Tables_4-5.zip)

Zip file is available for download at https://doi.org/10.5281/zenodo.10251085
